# Assessment of Trace Metals in *Camelus dromedarius* Meat from Mauritania

**DOI:** 10.1007/s12011-022-03144-3

**Published:** 2022-02-10

**Authors:** El Boukhary Ahmed, Mohamed Salem El Mahmoud Hamed, Babah Sidi Moktar, Angelo Santana-Del Pino, Mohamed Brahim, Mariem Youssouf Issa, Mohamed Lemine Zamel, Sarah Montesdeoca-Esponda

**Affiliations:** 1 Ministère de Santé Mauritania, Nouakchott, Mauritania; 2 Département Chimie Microbiologie Et Suivi du Milieu Aquatique (DCM-SMA), Office National d’inspection Sanitaire Des Produits de La Pêche Et de L’Aquaculture (ONISPA), 1416 Nouadhibou, Mauritania; 3 Département Biologie, Faculté Des Sciences Et Techniques (FST), Université de Nouakchott Al-Aasriya, Campus Universitaire de Nouakchott, 880, Route de Nouadhibou, Nouakchott, Mauritania; 4 Departamento de Matemáticas, Universidad de Las Palmas de Gran Canaria, Campus Universitario de Tafira, s/n, 35017 Las Palmas de Gran Canaria, Spain; 5 Institut Supérieur de Biotechnologie de Sfax, Université de Sfax-Tunisie, Sfax, Tunisia; 6 Instituto de Estudios Ambientales Y Recursos Naturales (I-UNAT), Universidad de Las Palmas de Gran Canaria, 35017 Las Palmas de Gran Canaria, Spain

**Keywords:** Trace metals, Camel meat, Dromedary meat, Mauritania, *Camelus dromedaries*

## Abstract

In Mauritania, the dromedary breeding is the most widespread pastoral activity, and it is considered as the first source of animal protein; however, the research on meat is relatively rare compared with fish, especially in relation with the presence of trace metals. In this work, livers, kidneys, and muscles of 25 *Camelus dromedarius* were collected from butcheries in Nouakchott (Mauritania) between February and April 2020 to study the concentration of trace metals (three essential metals: cooper (Cu), iron (Fe), and zinc (Zn), and four toxic metals: mercury (Hg), arsenic (As), cadmium (Cd), and lead (Pb)). Statistical treatment did not show significant differences associated with age (*P* = 0.7004), sex (*P* = 0.9353), or type of diet (0.9951) in the found concentration of the target substances, but the differences were significant between metals and between organs (*P* < 0.0001). The mean concentrations of the essential metals were 0.80 mg/100 g for Cu, 6.02 mg/100 g for Fe, and 3.28 mg/100 g for Zn, and the ratios between these concentrations were significant (*P* < 0.0001 in all cases), with [Fe] > [Zn] > [Cu]. Cu was most concentrated in the liver, Fe in the kidney, and Zn in muscle. The mean concentrations of toxic metals were 0.055 mg/kg for As, 0.064 mg/kg for Cd, 0.040 mg/kg for Pb, and 0.027 mg/kg for Hg. They also exhibited significant difference between organs. Hg and Pb showed their largest concentrations in the liver, whereas As and Cd reached their maximum values in the kidney. Therefore, the found concentrations in all cases were lower than the admissible level of trace metals.

## Introduction

Dromedary and camel meat are rich in essentials amino acids, and it is considered as the first source of animal protein [1]. This meat, with its good carcass yield and the dietary quality of its meat, is appreciated and consumed on a large scale in Mauritania as well as in West Africa and Middle East countries [2–4].

Mauritania is classified among the countries where the number of dromedaries is growing rapidly [1] and their meat consumption has increased recently. Thus, the dromedary breeding is the most widespread pastoral activity in the country, with a number of herds which are estimated as the most important of the countries of North, West, and Central Africa and as the third at the global level [2, 3].

However, dromedaries, like other animals, are exposed to many sources of pollution, which are enormous, and anthropogenic contaminants may accumulate in its meat. For example, some trace metals are known as toxic substances and they can cause serious disorders [5] in the short or long term [6], so, evaluation of its levels in animal’s organs allows us to inquire into the chemical quality of the food and the environment. When these elements exist in animal feed, they are accumulated in the kidneys and livers [7, 8]; therefore, these organs represent a significant source of exogenous dietary intake of trace elements when they are consumed by humans [9].

The presence of different metals has been studied in camels and dromedaries, and concentrations up to hundreds of mg/kg were found in several matrices, such as meat [6, 10–14], organs [4, 12, 15–17], blood [18–21], or milk [22] as shown in Table 1.

**Table 1:** Metal concentrations found in different matrices from camel

Sample	Metals	Location	Concentration range (mg/kg)	Ref
Liver, kidney, muscle	Cu, Zn, Fe, Hg, As, Cd, Pb	Mauritania	Cu: 1.80–68.2 Zn: 9.7–77.9 Fe: 19.8–167.2 Hg: 0.049–0.0935 As: < 0.0001–0.4606 Cd: < 0.0001–1.0483 Pb: 0.0164–0.5408	This work
Liver, kidney, muscle	Pb, Cd, As, Cu, Zn, Fe	Saudi Arabia	Pb: 0.00011–0.00395 Cd: 0.00112–0.01523 As: 0.0074–0.09995 Cu: 0.00006–0.00502 Zn: 0.22–6.77 Fe: 2.14–20.98	[4]
Sausages	Cr, Cu, Mn, Zn, Ni, Fe	Sudan	Cr: 0.36 Cu: 0.02 Mn: 0.04 Zn: 0.16 Ni: 0.01 Fe: 37.81	[6]
Meat	Fe, Mn, Cu, Zn, Pb, Cd, and Hg	Saudi Arabia	Fe: 70.98–113.07 Mn: 0.82–2.80 Cu: 1.33–3.20 Zn: 16.74–40.17 Pb: 2.01–5.48 Cd: 0.83–1.07 Hg: 0.024–0.054	[10]
Meat	Fe, Cu, Zn, Pb, Cd, Hg	Algeria	Fe: 70.98–75.03 Cu: 2.20–2.82 Zn: 23.51–40.17 Pb: 2.01–3.21 Cd: 0.83–0.91 Hg: 0.024–0.032	[11]
Kidney, liver, muscle, hide, blood	Pb, Cd, Cr	Nigeria	Pb: 0.11–1.17 Cd: 0.01–0.8 Cr: 0.13–0.59	[12]
Meat, liver, lung, heart, kidney	Co, Zn, Cd, Pb	Morocco	Co: 1.10–14.22 Zn: 4.05–10.88 Cd: 0.023–0.69 Pb: 0.71–1.33	[13]
Meat	Zn, Fe, Cu	Algeria	Zn: 140.56 Fe: 61 Cu:13.22	[14]
Liver, kidney, muscle	Cd, Zn, Cu, Co	Iraq	Cd: 2.961–4.191 Zn: 28.347–138.221 Cu: 4.663–26.754 Co: 1.913–8.194	[15]
Liver, muscle	As, Cd, Cr, Pb, Cu, Fe, Mn, Zn, Co	Iran	As: 0.100–1.292 Cd: 0.012–0.029 Cr: 2.333–9.930 Pb: 0.093–1.563 Cu: 1.220–4.381 Fe: 38.088–101.927 Mn: 0.301–1.293 Zn: 23.254–171.463 Co: 0.006–0.995	[16]
Liver, kidney, muscle	Pb, Cd, Hg, Cu, Zn	Egypt	Pb: 0.17–0.49 Cd: 0.03–0.12 Hg: 0.39–1.19 Cu: 0.10–8.82 Zn: 3.25–8.35	[17]
Blood serum	Fe, Zn, Cu, Cd, Mo, Se, Mn, Pb	USA	Fe: 2448.2* Zn: 274* Cu: 198.7* Cd: 89.8* Mo: 12.3* Se: 10.5* Mn: 0.74* Pb: 0.17*	[18]
Seminal plasma	Cd	USA	50–240*	[19]
Blood	Cu, Se	Saudi Arabia	Cu: 58.6–70.3 Se: 4.6–5.3	[20]
Blood	Zn, Cu	Sudan	Zn: 597–813 Cu: 51.0–67.1	[21]
Milk	Cd, Pb, Hg	Iran	Cd: 0.000006–0.00425 Pb: 0.00012–0.01674 Hg: 0.00103–0.00561	[22]
Neck, shoulder, plate, leg and loin	Mg, Pb, Fe, Hg	Iraq	Mg: 7.50–26.2 Pb: 1.3–5.5 Fe: 35.0–350.0 Hg: 0.0011–0.0045	[23]
Liver, muscle	Pb, Cd, As	Saudi Arabia	Pb: 0.0073–0.0105 Cd: 0.0009–0.0013 As: 0.008–0.0116	[24]

Most of these studies have been carried out in countries where camel is an important food source for the population (Iran, Saudi Arabia, Algeria, Nigeria, Morocco, Sudan, Iraq), so the presence of certain trace metals could affect the quality of their nutritional properties to humans. Hence, it is important to know the content of these elements at the same time that the food nutritional studies are conducted.

However, in Mauritania, the research on meat is relatively scarce compared with fish, especially regarding the presence of toxic metals and health risk related. For the best of our knowledge, no studies have been performed in Mauritania to estimate the concentration levels of metals present in camel or dromedary meat.

Mauritania is known by the iron exploitation. Its production of this metal in 2020 was 12.5 billion tons [25]. Recently, the country has begun the exploitation of other metals such as copper and gold in the region of Inchiri, where the dromedaries in Mauritania go looking for pasture. These exploitations may have an impact on environment especially plant which is eaten by animals.

Thus, the main object of this work was to study some trace metals levels in dromedary meat from butcheries of Nouakchott city. The target essential metals were cooper (Cu), iron (Fe), and zinc (Zn), while the selected target toxic metals were mercury (Hg), arsenic (As), cadmium (Cd), and lead (Pb). A statistical analysis was conducted to know if the differences found between the metal concentrations were significantly related with the studied organs, and with the age, type of diet, or sex of the animals. The measured concentration levels of target analytes were also compared with studies analyzing camel and dromedary meat from other regions.

## Experimental

### Sampling

Dromedary meat was collected randomly from some butcheries located in Nouakchott city (Mauritania) between February and April 2020. Just after sampling, the samples were transported to the laboratory under ice. A total of 25 animals were dissected to separate their livers, kidneys, and muscles. These animals were classified as the following: 9 young (< 3 years old), 7 adults (4–5 years old), and 9 old (10 years old). In each age’s class, three animals were fed on natural food and the others were fed on forage (crops that have been cut and dried to be used to feed animals). The origin of animals could not be identified for some of them; however, the majority came from Inchiri region which is close to Nouakchott.

### Sample Preparation and Analyses

At laboratory, individual samples (20 ± 3 g of liver and kidney and 45 ± 5 g of muscle) were separately dried to constant weight at − 46 °C for 48 h with a lyophilizer system in acid-washed flask. Moisture content was calculated and samples were ground to a fine powder using a porcelain mortar and a pestle.

Samples of 0.2-g dry weight (dw) were digested by duplicate according to El Mahmoud et al. [26]. and Taweel et al. [27]. Briefly, samples were placed in microwave-closed vessels with a mixture of ultra-pure nitric acid 67% and hydrogen peroxide 30% (3:1) [28] at room temperature for 1 h. Digestion conditions applied in the microwave system were as follows: 3 min at 250 W; 5 min at 650 W; 22 min at 500 W; and finally 5 min at 0 W and vent. Hydrogen peroxide with nitric acid was added to the samples because peroxide decreases nitrous vapors and speeds up the digestion of organic substances by elevating the reaction temperature [29]. Then the digested samples were diluted to 20 mL with deionized water (milli-Q quality).

The trace metal analysis, except for Hg, was conducted using Indicative Plasma Couple-optic emission specter (ICP-OES), which limit of detection (LOD) is 0.001 mg/kg. Hg was analyzed using Direct Mercury Analyzer (LOD = 0.003 mg/kg) according to the method “MA.207–Hg 2.0. Rév. 4. Centre d’expertise en analyse environnementale du Québec CEAEQ” [30]. Standard solution and *r*-square of the calibration curves are shown in Table 2.

**Table 2:** Standard concentrations and *r*-square of the calibration curves

Standards	1	2	3	4	5	6	*r*-square
Metals							
Cu (mg/l)	0.1	0.5	1	2	5	10	0.9960
Zn (mg/l)	0.1	0.5	1	2	5	10	0.9990
Fe (mg/l)	0.1	0.5	1	2	5	10	0.9961
As (mg/l)	0.1	0.5	1	2	5	10	0.9994
Cd (mg/l)	0.1	0.5	1	2	5	10	0.9967
Pb (mg/l)	0.1	0.5	1	2	5	10	0.9976
Hg (ng)	10	20	50	100	150	200	0.9998

### Statistical Analyses

Concentrations of each metal were initially described as mean ± standard deviation (sd) for every organ, age group, and type of diet (natural or forage). As data were collected several times for the same individual (in liver, kidney, and muscle), linear mixed effects models have been used to evaluate the significance of the differences between the concentrations of the various metals as well as the differences in metal concentrations between organs, adjusting for the effects of age, type of diet, and sex, and taking into account the random effect of the individual. When significant differences were detected, Tukey post hoc tests were performed to identify between which specific organs differences occurred and to evaluate their magnitude. Metal concentrations were logarithmically transformed to meet the normality hypothesis. Normality was tested using the Shapiro–Wilk test. Due to this logarithmic transformation, the magnitude of the differences in metal concentration between different organs was expressed in proportional terms (ratios of concentrations), accompanied by their corresponding 95% confidence intervals. A significance level of 5% was used for all contrasts. General statistical analysis and figures were performed using R software version 4.0.1 [31]. Mixed model fitting was performed using also in R the nlme package [32], and adjusted means were evaluated using the emmeans package [33].

## Results

Means and SD of metal concentrations found in meat samples, separated according to age group and type of diet, are reported for essential metals (Cu, Zn, and Fe) and for toxic metals (Hg, Cd, As, and Pb) in Table 3.

**Table 3:** Metal concentrations in dromedary meat and SD

Age	Organ	Food	Essentials metals (mg/100 g)			Toxics metals (mg/kg)			
			Cu	Zn	Fe	Hg	As	Cd	Pb
0–3 years	Liver	Natural food	6.82 ± 2.45	4.26 ± 0.90	5.77 ± 2.47	0.0320 ± 0.0200	0.1094 ± 0.0899	0.1086 ± 0.0818	0.1420 ± 0.2460
		Animal feed	2.91 ± 3.16	2.73 ± 1.73	6.93 ± 4.30	0.0878 ± 0.0495	0.1684 ± 0.1779	0.2300 ± 0.2218	0.1407 ± 0.1514
	Muscle	Natural food	0.35 ± 0.38	7.79 ± 2.50	1.98 ± 1.07	0.0049 ± 0.0008	0.0145 ± 0.0157	0.0069 ± 0.0120	0.0164 ± 0.0283
		Animal feed	0.69 ± 0.89	5.62 ± 2.19	3.00 ± 0.95	0.0388 ± 0.0389	0.0059 ± 0.0145	0.0670 ± 0.1050	0.1693 ± 0.1994
	Kidney	Natural food	0.29 ± 0.13	1.85 ± 0.49	10.55 ± 3.34	0.0179 ± 0.0035	0.3634 ± 0.1281	0.4103 ± 0.1466	0.0242 ± 0.0420
		Animal feed	1.03 ± 1.44	2.10 ± 1.46	10.51 ± 3.29	0.0486 ± 0.0251	0.1001 ± 0.0819	0.1491 ± 0.1295	0.2232 ± 0.4459
4–5 years	Liver	Natural food	5.75 ± 2.41	2.99 ± 0.86	8.26 ± 0.72	0.0266 ± 0.0074	0.2912 ± 0.4232	0.0313 ± 0.0302	0.1614 ± 0.2652
		Animal feed	3.61 ± 2.98	2.84 ± 0.73	10.23 ± 3.76	0.0935 ± 0.0338	0.4606 ± 0.3395	0.3564 ± 0.3304	0.1060 ± 0.2120
	Muscle	Natural food	0.55 ± 0.43	7.32 ± 3.38	2.17 ± 1.55	0.0093 ± 0.0100	< 0.0001	< 0.0001	0.0461 ± 0.0799
		Animal feed	1.58 ± 2.30	4.72 ± 2.32	4.29 ± 1.69	0.0555 ± 0.0274	< 0.0001	0.1858 ± 0.1839	0.0349 ± 0.0699
	Kidney	Natural food	0.39 ± 0.18	1.73 ± 0.39	12.74 ± 0.04	0.0251 ± 0.0109	0.3922 ± 0.3716	0.1522 ± 0.1866	0.0977 ± 0.0847
		Animal feed	0.57 ± 0.20	2.73 ± 2.68	12.70 ± 6.20	0.0538 ± 0.0257	0.3142 ± 0.4498	1.0483 ± 1.2258	0.2244 ± 0.3374
10 years	Liver	Natural food	3.49 ± 3.94	3.13 ± 2.59	5.09 ± 3.45	0.0377 ± 0.0058	0.0698 ± 0.0259	0.0524 ± 0.0255	0.1648 ± 0.0338
		Animal feed	4.73 ± 3.20	3.68 ± 0.77	6.34 ± 1.45	0.0528 ± 0.0269	0.1450 ± 0.0628	0.2024 ± 0.0755	0.2188 ± 0.2016
	Muscle	Natural food	0.62 ± 0.72	6.10 ± 3.25	5.10 ± 3.53	0.0085 ± 0.0063	0.0175 ± 0.0163	0.0135 ± 0.0234	0.1691 ± 0.1757
		Animal feed	0.33 ± 0.18	5.99 ± 2.64	4.13 ± 3.11	0.0119 ± 0.0111	0.1181 ± 0.1620	0.0896 ± 0.1701	0.5408 ± 1.1439
	Kidney	Natural food	0.18 ± 0.25	0.97 ± 1.37	16.72 ± 0.01	0.0268 ± 0.0138	0.3145 ± 0.4448	0.3594 ± 0.5083	0.0849 ± 0.1200
		Animal feed	0.82 ± 1.00	2.70 ± 2.40	9.34 ± 5.45	0.0237 ± 0.0144	0.3107 ± 0.2360	0.3010 ± 0.2890	0.0660 ± 0.0946

Moisture content varied widely between the liver and other organs. It was 65.3 ± 3.65%, 75.69 ± 3.32%, and 79.95 ± 3.49% for the liver, muscle, and kidney, respectively. These results are similar to other found in camel meat from Algeria (Ouargla), containing 76 ± 0.8% of moisture [14] while Hammad et al. reported values of 74.72 ± 2.45% for camel meat from Sudan [34].

### Essentials Metals

No significant differences associated with age (*P* = 0.7004), sex (*P* = 0.9353), or type of diet (0.9951) were detected in the concentration of any of the analyzed metals, but the differences were significant between metals (*P* < 0.0001) and between organs (*P* < 0.0001) as well as in the interaction between both variables. When adjusted by age, type of diet, and sex, and averaging across organs, the mean concentrations of essential metals were 0.80 mg/100 g (*CI* 95% [0.68, 0.94]) for Cu, 6.02 mg/100 g (*CI* 95% [5.06, 7.14]) for Fe, and 3.28 mg/100 g (*CI* 95% [2.79, 3.86]) for Zn. Table 4 shows the ratios of the adjusted mean concentrations between these metals as well as their corresponding 95% confidence intervals and the *P*-values for testing if these ratios are equal to one. It can be seen that all ratios between these concentrations were significant (*P* < 0.0001 in all cases), with [Fe] > [Zn] > [Cu].

**Table 4:** Ratios between adjusted mean concentrations of metals

Type	Metals	Ratio	*CI* 95%	*P*
Essential	Cu/Fe	0.134	[0.106, 0.171]	< 0.0001
	Cu/Zn	0.246	[0.195, 0.310]	< 0.0001
	Fe/Zn	1.832	[1.447, 2.318]	< 0.0001
Toxic	Ar/Cd	0.889	[0.520, 1.520]	0.9423
	Ar/Pb	1.302	[0.762, 2.224]	0.5819
	Ar/Hg	1.753	[1.026, 2.997]	0.0360
	Cd/Pb	1.463	[0.856, 2.501]	0.2586
	Cd/Hg	1.971	[1.153, 3.369]	0.0066
	Pb/Hg	1.347	[0.788, 2.302]	0.4771

**Fig. 1:**
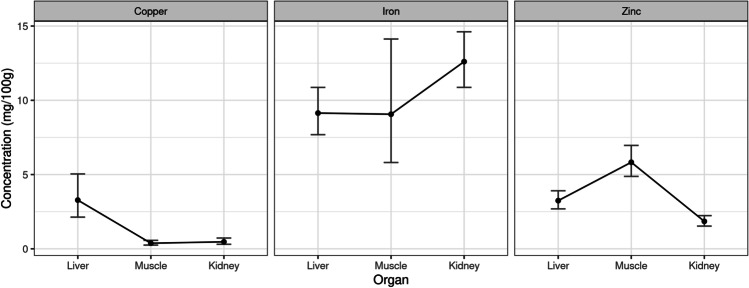
Adjusted mean and 95% confidence interval for concentration of essential metals by organ

Nevertheless, these concentrations were not uniform through the various organs. Essentials metal (Cu, Zn, and Fe) concentrations showed a variable level in the different organs. Table 5 shows the adjusted mean concentrations (with 95% confidence intervals) of each essential metal in the various organs. These means and confidence intervals are depicted in Fig. 1, where it can be seen that, apparently, Cu is most concentrated in the liver, Fe in the kidney, and Zn in muscle. Table 6 shows the ratios between these concentrations.

**Table 5:** Adjusted mean and 95% confidence interval for concentration of metals by organ

Type	Metal	Liver	Muscle	Kidney
Essential	Cu	3.279 [2.132, 5.041]	0.377 [0.249, 0.569]	0.470 [0.304, 0.725]
	Zn	3.243 [2.690, 3.908]	5.826 [4.875, 6.962]	1.848 [1.529, 2.233]
	Fe	9.140 [7.686, 10.869]	9.062 [5.812, 14.126]	12.604 [10.870, 14.614]
Toxic	Hg	0.044 [0.030, 0.065]	0.010 [0.007, 0.015]	0.024 [0.017, 0.035]
	Ar	0.116 [0.062, 0.210]	0.009 [0.001, 0.023]	0.134 [0.073, 0.240]
	Cd	0.091 [0.044, 0.178]	0.015 [0.004, 0.036]	0.126 [0.063, 0.243]
	Pb	0.054 [0.023, 0.118]	0.030 [0.010, 0.068]	0.028 [0.009, 0.065]

**Table 6:** Ratios of concentration of metals between organs. For each ratio, a 95% confidence interval is shown, as well as a *p*-value for testing its significance

Type	Metal	Liver/muscle	Liver/kidney	Muscle/kidney
Essential	Cu	8.71 [4.62, 16.41] (P < .0001)	6.98 [3.65, 13.33] (P < .0001)	0.80 [0.43, 1.51] (P = 0.6770)
	Zn	0.56 [0.41, 0.75] (P < .0001)	1.75 [1.29, 2.37] (P = 0.0002)	3.14 [2.34, 4.22] (P < .0001)
	Fe	1.01 [0.55, 1.86] (*P* = 0.9991)	0.73 [0.54, 0.97] (*P* = 0.0345)	0.72 [0.40, 1.30] (*P* = 0.2909)
Toxic	Hg	4.23 [2.68, 6.66] (*P* < 0.0001)	1.82 [1.15, 2.89] (*P* = 0.0078)	0.43 [0.27, 0.68] (*P* = 0.0001)
	Ar	6.50 [2.69, 15.67] (*P* < 0.0001)	0.88 [0.36, 2.13] (*P* = 0.9316)	0.13 [0.06, 0.32] (*P* < 0.0001)
	Cd	4.05 [1.51, 10.87] (*P* = 0.0036)	0.74 [0.27, 2.02] (*P* = 0.7541)	0.18 [0.07, 0.49] (*P* = 0.0004)
	Pb	1.64 [0.78, 3.42] (*P* = 0.2487)	1.71 [0.81, 3.62] (*P* = 0.2041)	1.05 [0.50, 2.20] (*P* = 0.9886)

The mean concentration of Cu in the liver is 8.71 times greater than in muscle and 6.98 times greater than in the kidney. Both differences are significant (*P* < 0.0001). However, the mean value of the concentration of Cu in muscle is only the 80% of the value in the kidney, this difference being not significant (*P* = 0.6770). So, Cu concentration in the organs follows the order liver > muscle = kidney.

With respect to Zn, the adjusted mean concentration of this metal (Table 5) in the liver is 56% of the mean concentration in muscle and 1.75 times greater than in the kidney. Also, the mean concentration in muscle is 3.14 times higher than the one found in the kidney, being all the differences significant (*P* < 0.0002). So, Zn concentration in the organs follows the order muscle > liver > kidney.

Regarding the analysis of Fe, the mean concentration (Table 6) in the liver is almost the same as in muscle (*P* = 0.9991), and is 73% of the concentration in the kidney, being this difference significant (*P* = 0.0345). Mean concentration in muscle is 72% of the concentration in the kidney but this difference is not significant (*P* = 0.2909). So, for Fe, the concentration in organs follows the order liver < kidney, whereas muscle has no significant differences with any of those two organs.

### Toxic Metals

As with essential metals, the application of the linear mixed model did not detect any significant effects associated to age (*P* = 0.6375), type of diet (*P* = 0.0744), or sex (*P* = 0.8523), but there were significant differences in the concentrations between metals (*P* = 0.0074) and organs (*P* < 0.0001), as well as in its interaction (*P* = 0.0004). The adjusted mean concentrations were 0.055 mg/kg (*CI* 95% [0.037, 0.081]) for As, 0.064 mg/kg (*CI* 95% [0.043, 0.093] for Cd, 0.040 mg/kg (*CI* 95% [0.026, 0.060] for Pb, and 0.027 mg/kg (*CI* 95% [0.017, 0.042] for Hg. When global concentrations of toxic metals are compared (Table 3), the concentration of Hg is significantly less than As (*P* = 0.036) and Cd (*P* = 0.0066), but has no significant difference with Pb (*P* = 0.4771); As, Cd, and Pb have no significant differences in their respective global concentrations.

**Fig. 2:**
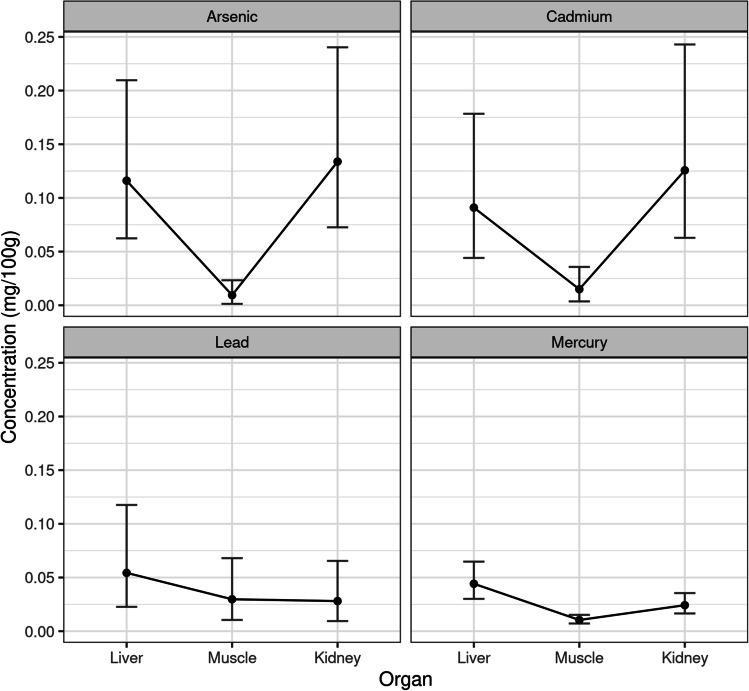
Adjusted mean and 95% confidence interval for concentration of toxic metals by organ

Toxic metals also showed significant differences in their concentrations, in the various organ (*P* = 0.0004). Table 5 shows the adjusted means and a 95% confidence interval for the concentration of toxic metals in the different organs. Figure 2 represents these means and confidence intervals.

Hg and Pb show their highest concentrations in the liver, whereas As and Cd reach their largest values in the kidney. Ratios of adjusted concentrations for toxic metals between the organs and 95% confidence intervals, as well as *P*-values for testing the significance of the ratios, are shown in Table 6. It can be seen that adjusted mean concentration of Hg in the liver is 4.23 times greater than in muscle (*P* < 0.0001) and 1.82 times greater than in the kidney (*P* = 0.0078). In muscle, the mean concentration of Hg is 43% of the concentration in the kidney (*P* = 0.0001). So, for Hg, the concentrations follow the order liver > kidney > muscle.

We also see that mean concentration of As in the liver is 6.5 times greater than in muscle (*P* < 0.0001), but shows no significant difference with the kidney (*P* = 0.9316). Also, mean concentration in muscle is only 13% of the mean concentration in the kidney (*P* < 0.0001). So, the concentrations of As in the organs follow the order liver = kidney > muscle.

The adjusted mean concentration of Cd in the liver is 4.05 times greater than in muscle (*P* = 0.0036) but shows no significant difference with the kidney (*P* = 0.7541). Also, mean concentration in muscle is only 18% of the mean concentration in the kidney (*P* < 0.0001).

So, the concentrations of Cd in the organs are liver = kidney > muscle. Lastly, Pb does not show significant differences between any of the organs.

## Discussion

### Essentials Metals

The results of the present study for Cu content are in accordance with those obtained by Chafik et al. (2004) in Morocco, where the liver appeared as the organ with highest presence of this metal [35]. Cu is stored by the liver and excreted into the bile, but some inherited defects in copper metabolism result in chronic accumulation that eventually causes hepatitis, hepatic failure, cirrhosis, and ultimately death [36]. Since it has been demonstrated that the level of Cu in organs like the liver is depending on the content in diet [37], more information about feeding locations will be useful to obtain comparable information. Cu level in the edible tissues of the dromedary from our study (Table 3) was also similar to the level reported by other authors [4, 10, 11, 14].

Regarding the ages of the camels, Sahraoui et al. reported that Cu content in dromedary meat from Algeria was almost the same for different age ranges (0.31 ± 0.1 mg/100 g for < 3 years old and 0.32 ± 0.08 mg/100 g for 4–6 years old individuals) [14], while our results showed an increase of Cu concentrations for 4–5 year-old animals regarding those 0–3 years old and olds ones. Although Cu content in edible tissues of camel from Saudi Arabia was also comparable with ours (ranging from 0.006 to 0.502 mg/100 g), concentration in the liver was lower (0.166 ± 0.092 mg/100 g) than ours [4]. Moreover, two studies, performed in Sudan, reported Cu concentration in meat lower than ours (0.002 ± 0.0050 mg/100 g [6] and 0.16 mg/100 g [34]).

The range of Zn concentrations measured in this study was comparable to those found in the literature for different edible parts of camel [11, 13, 16]. Zn concentration found in camel meat from Algeria was 3.3 mg/100 g [14], while Hammad et al. (2019) reported that camel meat from Sudan contained 3.49 ± 0.002 mg/100 g of Zn [34]. However, other authors found a very low concentration in meat of camel from Sudan (0.066 mg Zn/100 g) [6]. Zn content in edible tissues of camel from Saudi Arabia was also much lower than the one found in our study, in the ranges 0.133–0.355 mg/100 g in the kidney, 0.307–0.677 mg/100 g in the liver, and 0.022–0.199 mg/100 g in muscle [4]. In other study carried out in Morocco, muscle and the liver were the organs that accumulated the highest concentration of Zn [35]. The higher presence of Zn in the muscle than in other studied samples is related with its importance for skeletal muscle performance and resistance to fatigue [38], and it performs important roles in regulating whole-body metabolism [39].

This study showed Fe levels similar with findings in other animals from the same region, such as Algeria [11, 14] or more distant areas like Saudi Arabia [10], Iran [16], Iraq [23], or Sudan [6]. In detail, Sahraoui et al. (2018) from Algeria samples reported that Fe concentration is 6.10 ± 0.45 mg/100 g in camel meat [14], while camel sausages from Sudan contained 3.78 ± 0.04 mg/100 g of Fe [6]; both of them are in the same range of our results. However, higher values were found in camel muscle from that country (45.5 mg/100 g) [25]. Regarding other body parts, El-Ghareeb et al. (2019) reported the following concentrations in camel from Saudi Arabia: 1.82 ± 0.22 mg/100 g in the liver and 1.32 ± 0.25 mg/100 g in the kidney, which were lower than the outcomes of this work (up to 6 and 12 times for the liver and kidney, respectively) [4]. Some authors suggest that the different metabolism mechanisms occurred in the kidney are modulated directly or indirectly, among other factors, by cellular iron content, and they pointed out the treatment of iron-induced kidney injury by customized iron removal or relocation [40].

Given that some authors stated that the presence of some trace metals is clearly linked to diet composition, their levels can be related with both pasture and drinking water [38]. Most animals of this study come from Inchiri (Mauritania) which is an area known by the presence of metal exploitations, especially Cu, Fe, and gold. So, the level of these metal in dromedary meat could be related with this activity.

### Toxic Metals

Toxic metals are non-essential elements, and they can be accumulated in body causing diseases, especially nerve’s system problems. The results show that metals are more concentrated in the liver than in other studied parts of the camels which is linked with its biological role; the liver becomes the organ most affected by trace metal accumulation. So, these results are like the provided by other studies.

Hg content in edible camel parts was comparable with those measured in Saudi Arabia by Alturiqui et al. (0.024–0.054 mg/kg [10]), but higher than those found in camel from Algeria (0.006 ± 0.002 mg/kg [23]) and Iraq (0.0011–0.0045 mg/kg [39]).

Pb concentration in camel meat from Mauritania was lower than the value measured in Algeria (0.64 ± 0.04 mg/kg [23]), Saudi Arabia [10], and Iraq [23]. The high concentration of Pb in the liver also agrees with the results obtained by Abdelbasset et al., which confirm the previously mentioned trend of this organ to accumulate some metals like Pb [41].

As and Cd concentrations in the kidney and liver did not show significant variation probably because of kidney biological role, since these metals are cleaned from blood by the kidney in order to be excreted in urine.

The level of As in camel meat of this study is similar to these obtained by Meligy et al. in Saudi Arabia camel meat [24], but lower than these reported by Asli et al. from Iran [16].

Concentrations of Cd are lower than the results reported by authors from Algeria (0.116 ± 0.07 mg/kg), Saudi Arabia, and Iran [16, 24, 42].

The authors, except El-Ghareeb et al. from Saudi Arabia [4], found that these toxic metals (Cd, Pb, and As) were more concentrated in the liver than in the kidney and muscle [16, 23, 34, 39].

## Conclusions and Future Trends

The dromedary meat is widely consumed in Mauritania, so the control of their quality regarding the presence of pollutants is essential for the development and maintenance of the breeding activity and citizen health care. The results of this study allowed to determine essential and toxic metal concentrations in samples of the dromedaries.

No significant differences associated with age, sex, or type of diet were detected in the found concentrations for the studied essentials metals. However, they were significant between metals and organs, as well as in the interaction between both variables. The highest concentrations were found for Fe, followed by Zn, while the lowest levels were measured for Cu. Moreover, it was noticed that Cu was the most concentrated metal in the liver, Fe in the kidney, and Zn in muscle. Regarding the target toxic metals, found differences in the measured concentrations were not significant with respect to age, type of diet, or sex, but they were significant between metals and organs, as well as in its interaction. For this group of metals, the highest concentrations of Hg and Pb were found in the liver, while As and Cd were highest in the kidney.

Given that the concentrations of several metals in the different organs seem to be dependent on the content in diet, future works must be focused to offer information about meat origin. It would be interesting to know if location is a variable with statistical significance in the occurrence of metal traces, and from this starting point, design a study to investigate the differences between the quality of food among several locations.

Although in general, the obtained results are comparable with those reported by works carried out in Africa, it is necessary to carefully observe the levels of metals in edible parts of camels and dromedaries, especially toxic ones, since they could be dangerous when the consume of this meat by population is frequent.

The findings of this study can be used as reference of trace metal survey in meat, and the new information could be added to the available ones about the concentration data in other food, such as fish and chicken, to assess Mauritania citizen daily intake of trace metal.

## Data Availability

The authors confirm that all relevant data are included in the article.
